# Proteomic Analysis Reveals the Deregulation of Inflammation-Related Proteins in Acupuncture-Treated Rats with Asthma Onset

**DOI:** 10.1155/2012/850512

**Published:** 2012-11-14

**Authors:** Yu-Dong Xu, Jian-Mei Cui, Yu Wang, Lei-Miao Yin, Chang-Ke Gao, Xiao-Yan Liu, Ying Wei, Yan-Yan Liu, Yong-Liang Jiang, Chun-Xiao Shan, Yong-Qing Yang

**Affiliations:** ^1^Molecular Biology Laboratory, Shanghai Research Institute of Acupuncture and Meridian, Shanghai University of Traditional Chinese Medicine, Shanghai 200030, China; ^2^Yue Yang Hospital, Shanghai University of Traditional Chinese Medicine, Shanghai 200437, China

## Abstract

Although the beneficial effects of acupuncture in asthma treatment have been well documented, little is known regarding the biological basis of this treatment. Changes in the lung proteome of acupuncture-treated rats with asthma onset were comparatively analyzed using a two-dimensional gel electrophoresis (2DE) and mass-spectrometry- (MS-) based proteomic approach. Acupuncture on specific acupuncture points appeared to improve respiratory function and reduce the total number of leukocytes and eosinophils in bronchoalveolar lavage fluid in OVA-induced asthma onset. Image analysis of 2DE gels revealed 32 differentially expressed acupuncture-specific protein spots in asthma onset; 30 of which were successfully identified as 28 unique proteins using LC-MS/MS. Bioinformatic analyses indicated that these altered proteins are most likely involved in inflammation-related biological functions, and the functional associations of these proteins result in an inflammation signaling pathway. Acupuncture regulates the pathway at different levels by regulating several key nodal proteins, including downregulating of proinflammatory proteins (e.g., S100A8, RAGE, and S100A11) and upregulating of anti-inflammatory proteins (e.g., CC10, ANXA5, and sRAGE). These deregulated inflammation-related proteins may mediate, at least in part, the antiasthmatic effect of acupuncture. Further functional investigation of these acupuncture-specific effector proteins could identify new drug candidates for the prophylaxis and treatment of asthma.

## 1. Introduction

Asthmais a major public health problem that is characterized by an inflammatory disorder of the airways [1]. The global prevalence, morbidity, mortality, and economic burden associated with asthma have increased worldwide over the past two decades. Approximately 300 million people worldwide currently have asthma, and it is estimated that an additional 100 million people will be affected by 2025 [2]. Although current allopathic treatments, including anti-inflammatory and muscle-relaxing drugs, can provide temporary symptomatic relief, they do not prevent the natural course of asthma [3, 4]. Because of unsatisfactory pharmacological interventions and adverse drug reactions, many asthma sufferers turn to alternative or complementary therapies for improved asthma management [5]. 

Acupuncture is an effective therapy that has been practiced in China and other East Asian countries since 2000 B.C. This technique uses fine needles that are inserted into specific points of the body and manipulated with the intent of treating a wide variety of diseases. The World Health Organization endorses acupuncture for asthma and 42 other indications [6], and the US National Institutes of Health issued a consensus statement in 1997 proposing acupuncture as an effective treatment option for several health problems, including asthma [7]. We have previously reported that acupuncture treatment, given according to the principles of traditional Chinese medicine in patients with allergic asthma, has an immunomodulatory effect on inflammatory cells and cytokines and is associated with an improvement in general well-being [8], which is in good agreement with a study by Joos et al. [9]. Acupuncture can regulate the balance of the Thl/Th2 response and reduce leukotriene B4 and nitric oxide levels, which efficiently decreases immune-mediated lung inflammation in asthmatic rats [10, 11]. Although findings from clinical and experimental studies indicate that acupuncture has immunomodulatory and anti-inflammatory effects on asthma [12], little is known about the biological basis of acupuncture treatment. A global protein profiling study based on the effectiveness of acupuncture could reveal the molecular basis of acupuncture treatment and identify new therapeutic targets for asthma.

In recent years, a number of proteomic studies of acupuncture in neurological disorders, including neuropathic pain, Parkinson's disease, and spinal cord injury, have been published [13–15], and several candidate proteins associated with acupuncture effectiveness have been identified. However, few proteomic studies have been designed to investigate the effects of acupuncture on asthma. In this study, a 2DE/MS-based proteomic analysis of lung tissues from acupuncture-treated rats with OVA-induced asthma onset was performed to identify acupuncture-specific effector proteins, which are expressed as a result of asthma treatment, and elucidate the molecular mechanisms underlying the antiasthma effect of acupuncture.

## 2. Materials and Methods

### 2.1. Animals

All animal experiments and procedures have been approved by the Committee on the Ethics of Animal Experiments of Shanghai University of Traditional Chinese Medicine (approval ID: 08001) and were conducted in accordance with the regulations set forth by the State Science and Technology Commission. Pathogen-free, male Sprague-Dawley (SD) rats (four weeks old, 110–130 g, SLAC Laboratory Animal Co. Ltd.; Shanghai, China) were raised in a pathogen-free rodent facility and provided with food and water *ad libitum*. The rats were randomly divided into four groups: normal control rats (NC, *n* = 15), asthmatic rats (AS, *n* = 14), asthmatic rats treated with acupuncture (AA, *n* = 12), and normal rats treated with acupuncture (NA, *n* = 15). All rats were housed in animal facilities approved by the Shanghai Committee for the Accreditation of Laboratory Animals for at least one week before the experiments were initiated. 

### 2.2. Protocol for Sensitization and Allergen Challenge

**Figure 1:**
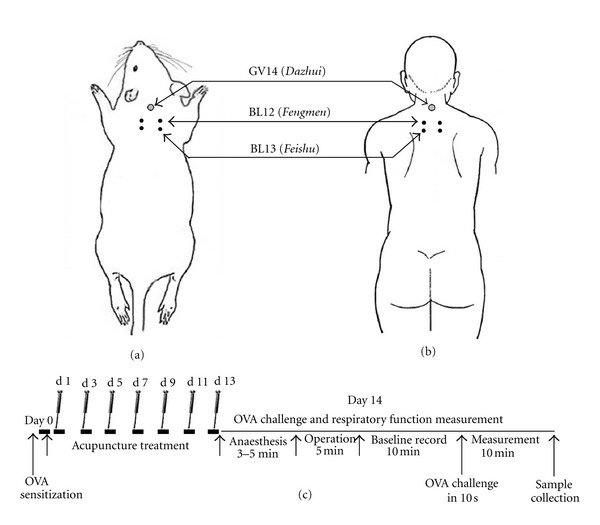
Schematic localization of acupuncture points: GV14 (*Dazhui*), BL12 (*Fengmen*), and BL13 (*Feishu*) stimulated in rats (a), and the corresponding equivalent acupuncture points in the human body (b). (c) Presentation of the experimental procedure; rats were sensitized with OVA at day 0. Acupuncture was repeatedly administered at specific acupuncture points on alternate days for 2 weeks from the first day after sensitization. On day 14, rats were anaesthetized and challenged with OVA by injection into the external jugular vein. The pulmonary resistance (RL), dynamic compliance (Cdyn), and respiratory rate (RR) were recorded before the challenge for 10 min as the baseline values and immediately measured for another 10 min after the OVA challenge. The sample collections were performed immediately after the measurements were taken. Control rats were sensitized and challenged with saline instead of OVA.

Rats were sensitized and challenged with ovalbumin (OVA, grade V, Sigma, Taufkirchen, Germany) according to a previously described protocol [17]. Briefly, on day 0, the rats were intraperitoneally injected with 1 mg of OVA precipitated with 10 mg of aluminum hydroxide gel and dissolved in 1 mL saline (0.9% NaCl). On day 14, allergic rats were anaesthetized with 1% sodium pentobarbitone (w/v) at a dose of 50 mg/kg by intraperitoneal injection and were challenged with 1 mL/kg of 5% OVA in saline (5 mg/kg) by injection into the external jugular vein over a 10 s period (Figure 1(c)). The rats in the NC and NA groups received the same treatment schedule but were sensitized and challenged with saline instead of OVA.

### 2.3. Acupuncture Treatment

The acupuncture points, GV 14 (*Dazhui*, between the C7 and T1 vertebrae), bilateral BL12 (*Fengmen*, foveola laterally between the T2 and T3 vertebrae), and bilateral BL13 (*Feishu*, foveola laterally between the T3 and T4 vertebrae), were selected based on the theory of traditional Chinese medicine in treating asthma [8] (Figure 1(a) and Figure 1(b)). In accordance with our clinical treatment of asthma [8], manual acupuncture was performed once every other day for two weeks beginning on the first day after sensitization (Figure 1(c)). Disposable stainless needles (13 mm long, 0.30 mm in diameter, Suzhou Medical Appliance Factory; Suzhou, China) were inserted through the skin to a depth of approximately 5 mm. The needles were twisted approximately 360° evenly at a rate of 60 times/min for 20 s, manipulated every 5 min, and withdrawn after 20 min. To conveniently manipulate the acupuncture points on the back, the rat was placed on a suspended shelf (50 × 45 mm, approximately 50 cm high from the ground, Figure Supplemental 1 available online at doi:10.1155/2012/850512), which calmed the rat and eliminated the need for anesthesia [16]. The same experienced practitioner performed all needle manipulations, and the animals were handled while awake. Special care was taken to minimize stress. The rats in NC and AS groups were also placed on the suspended shelf but did not receive acupuncture treatment.

### 2.4. Measurement of Pulmonary Function

Pulmonary function was assessed by measuring changes in the pulmonary resistance (RL), dynamic compliance (Cdyn), and respiratory rate (RR) in response to OVA challenge in anaesthetized, spontaneously breathing rats [16, 18]. Briefly, a rat was placed in a supine position and warmed with an incandescent lamp after anesthesia. At the upper part of the trachea, a T-shaped incision was made, and a T-shaped cannula, which was directly attached to a heater-controlled pneumotachograph (Fleisch model 000, Hans Rudolph; USA), was gently inserted into the trachea. Tidal flow was determined using a pneumotachograph connected to a differential pressure transducer (AutoTran, model 600D-011, ±2 cm H_2_O). To measure the transpulmonary pressure, a water-filled PE-90 tube was inserted into the esophagus to the mid-thorax level (lower one-third of the esophagus) and coupled to a pressure transducer (PT14MX, Jialong Teaching Equipment; Shanghai, China). The pneumotachograph tidal flow signal was integrated with respect to time to obtain the tidal volume. The pulmonary resistance (RL) and dynamic compliance (Cdyn) were calculated over a complete respiratory cycle using an integration method over flows, volumes, and pressures and were continuously recorded with software (Shanghai Medical College, Fudan University) designed for physiology experiments. The respiratory parameters were averaged in 60 s segments, and the maximum RL, minimum Cdyn, and change in respiratory rate (RR) values were taken and calculated as the differential value subtracted from the corresponding baseline value (Figure 1(c)). Experimental operators in lung function measurement, and the following BALF analysis, 2DE-MS/MS, and western blot analysis did not know the animal and sample allocation.

### 2.5. Bronchoalveolar Lavage Fluid (BALF) Collection and Analysis

BALF collection was performed immediately following the pulmonary function measurements. Each rat was anesthetized and the lung and heart were surgically exposed. The trachea was cannulated and the lung was lavaged three times with atotal volume of 10 mL of sterile saline. The recovered lavage fluid was pooled and centrifuged at 300 g for 10 min at 4°C. The cell pellet obtained from the BALF was resuspended in 100 *μ*L PBS containing 1% BSA, and the BALF total leukocyte and differential cell counts were performed using aHemavet950veterinary hematology system(Drew Scientific; Oxford, CT, USA).

### 2.6. Lung Tissue Sample Preparation

The excised right lobe of the lung was rinsed free of blood in ice-cold saline and immediately frozen in liquid nitrogen. Protein from each lung tissue was extracted using a ReadyPrep Sequential Extraction Kit (Bio-Rad; Hercules, CA) with PMSF (1 mM), DNase (RNase-free; 20 *μ*g/mL), and RNase (5 *μ*g/mL) added to Reagent 1 immediately before use. Protein concentrations were determined using a modified Bradford assay. For the 2DE analysis, protein extracts from animals in the same group were pooled equally according to protein quantity and stored at −80°C until use.

### 2.7. 2DE Analysis

Protein (100 *μ*g) was loaded onto 17 cm IPG strips with a linear separation range of pH 3–10 (Bio-Rad; Hercules, CA), which were subsequently rehydrated for 12 h at 50 V at 20°C. First-dimensional isoelectric focusing (IEF) was performed in a Protean IEF Cell (Bio-Rad). The IEF voltage was raised with a rapid ramp to 10,000 V and strips were run at 20°C with the current limited to 50 mA/strip, until reaching 60,000 Vh. After IEF, the focused IPG strips were equilibrated at room temperature in buffer (50 mM Tris-HCl pH 8.8, 6 M urea, 20% glycerol, and 2% SDS) with 2% DTT (w/v) for 15 min followed by a second equilibration in the same buffer containing 2.5% iodoacetamide (w/v) for 15 min. The strips were placed on 13% polyacrylamide gels and embedded in 1% agarose stacking gel. The second-dimensional SDS-PAGE was performed at 24 mA per gel until the bromophenol blue dye front reached the bottom of the gels. The gels were run in triplicate for each sample and stained with silver nitrate solution. For image analysis, the gels were scanned at a high resolution with a GS-800 densitometer (Bio-Rad), and PDQuest software version 7.1 (Bio-Rad) was used to detect altered protein expression levels. The automatic spot detection and matching of the gels were followed by manual validation of the matched and unmatched protein spots. The intensity volumes of individual spots were normalized with the total intensity volume of all spots present in each gel (%V). Only those protein spots with intensity alterations equal to or greater than twofold (*t*-test, *P* < 0.05) were considered to demonstrate significant differential expression.

### 2.8. In-Gel Digestion and LC-MS/MS Analysis

Protein spots with significant alterations in expression were manually excised from the 2DE gels, destained for 20 min with equal volumes of 30 mM potassium ferricyanide and 100 mM sodium thiosulfate at room temperature, and washed with Milli-Q water until the gels were destained. The spots were maintained in 0.2 M NH_4_HCO_3_ for 20 min before being lyophilized. Each spot was digested overnight with 12.5 ng/mL trypsin in 0.1 M NH_4_HCO_3_. The peptides were extracted three times with 50% CAN and 0.1% TFA. The identification of the digested proteins was conducted using a Finnigan LTQ mass spectrometer (ThermoQuest; San Jose, CA) coupled with a Surveyor HPLC system (ThermoQuest). First, a Microcore RP column (C18 0.15 mm × 120 mm; ThermoHypersil; San Jose, CA) was used to separate the protein digests. Solvent A was 0.1% formic acid, and solvent B was 0.1% formic acid in 99.9% ACN. The gradient was held at 2% solvent B for 15 min and linearly increased to 98% solvent B over 90 min. The peptides were eluted from a C18 microcapillary column at a flow rate of 150 *μ*L/min and electrosprayed directly into the LTQ mass spectrometer with an applied spray voltage of 3.2 kV. The scan ranged from m/z 400 to 2000. Protein identification using MS/MS raw data was performed using SEQUEST software (Thermo Finnigan) by searching against the Swiss-Prot rat protein database. The identification results were filtered using Xcorr (1+≥1.9, 2+≥2.2, 3+≥3.75) and DelCn (≥0.1).

### 2.9. Bioinformatic Analysis

To highlight the potential biological processes affected by acupuncture, differentially expressed proteins were classified into groups according to biological processes using the PANTHER (Protein ANalysis THrough Evolutionary Relationships) Classification System (http://www.pantherdb.org/). Protein classifications were reviewed, and minor changes were made based on published data regarding protein function (NCBI's GeneRIFs and/or protein data retrieval system iHOP). Proteins that did not match any entry in the PANTHER database or did not have an annotated function were assigned to one of PANTHER's parent groups, with the aid of the online Gene Ontology tool AmiGO (http://amigo.geneontology.org). Protein-protein interactions of proteins identified as having inflammatory biological functions were investigated using the STRING (Search Tool for the Retrieval of Interacting Genes/Proteins) database of physical and functional interactions [19]. 

### 2.10. Western Blot

The same protein samples used for 2DE were subjected to SDS-PAGE (12% to 15% polyacrylamide gels) and electrotransferred onto polyvinylidene fluoride membranes (Millipore; Billerica, MA). The membranes were blocked with PBS containing 5% nonfat milk and 0.1% Tween 20 for three hours at room temperature. The blocked membranes were incubated overnight at 4°C with specific primary antibodies against CC10 (1 : 5000, Millipore 07–623), RhoGDI2 (1 : 2000, Abcam ab14230), S100A8 (1 : 3000, Santa Cruz sc-8113), sRAGE (1 : 5000, R&D AF1616), and *β*-actin (1 : 5000, Abcam) and were washed and incubated with the appropriate horseradish-peroxidase- (HRP-) conjugated secondary antibodies for three hours at room temperature. Immunocomplexes were visualized using enhanced chemiluminescence detection reagents (Beyotime; Shanghai, China) on X-ray films. Proteins were quantified using optical density analysis of protein bands using Quantity One software (Bio-Rad). Each protein band was normalized to the corresponding *β*-actin band.

### 2.11. Statistical Analysis

Data were presented as the mean ± SEM. A one-way analysis of variance (ANOVA) was performed to evaluate the differences among the groups. Post hoc comparisons, if applicable, were performed using the Least Significant Difference (LSD) test. A value of *P* < 0.05 was considered statistically significant.

## 3. Results

### 3.1. Effect of Acupuncture on Respiratory Function Variability in Asthma Onset in Rats

**Figure 2:**
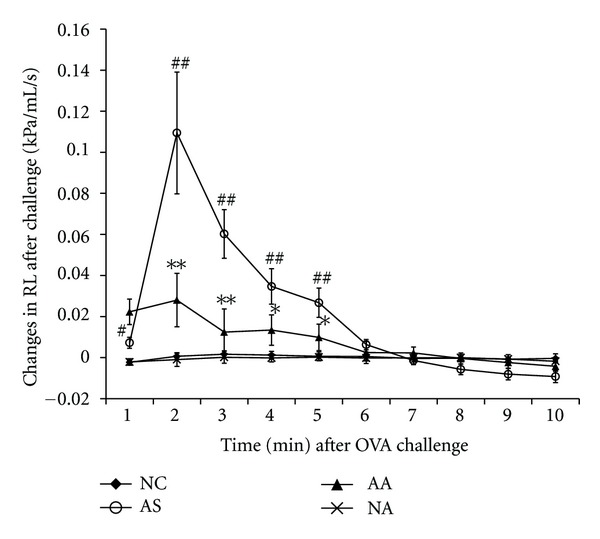
Changes in the pulmonary resistance (RL) within 10 min after challenge in NC (*n* = 15, sensitized and challenged with saline), AS (*n* = 14, sensitized and challenged with OVA), AA (*n* = 12, sensitized and challenged with OVA + acupuncture treatment) and NA (*n* = 15, sensitized and challenged with saline + acupuncture treatment).The changes in the RL are expressed as differential values subtracted from the corresponding baseline values (Figure 1). Each point represents the mean ± SEM. ^#^ *P* < 0.05, ^##^ *P* < 0.01 when comparing the AS group with the NC group and *P < 0.05, **P < 0.01 when comparing the AA group with the AS group.

The allergen challenge in OVA-sensitized rats induced a significant increase in the RL at 1 to 5 min (*P* < 0.05, Figure 2), and significant decreases in the simultaneously measured Cdyn and RR (Table Supplemental 1 available online at doi:10.1155/2012/850512) compared with control rats, thus indicating an OVA-induced asthma onset. When acupuncture was performed on the OVA-sensitized rats in the AA group, the RL was significantly decreased 2 to 5 min after the OVA challenge when compared with the AS group (*P* < 0.05, Figure 2), and Cdyn and RR were increased 2 to 5 min after the challenge when compared with the AS group (Table Supplemental 1 available online at doi:10.1155/2012/850512). In addition, in acupuncture-treated normal rats, RL was unchanged after saline challenge when compared with normal controls. 

### 3.2. Effect of Acupuncture on the Total Leukocyte and Differential Cell Counts in BALF

**Figure 3:**
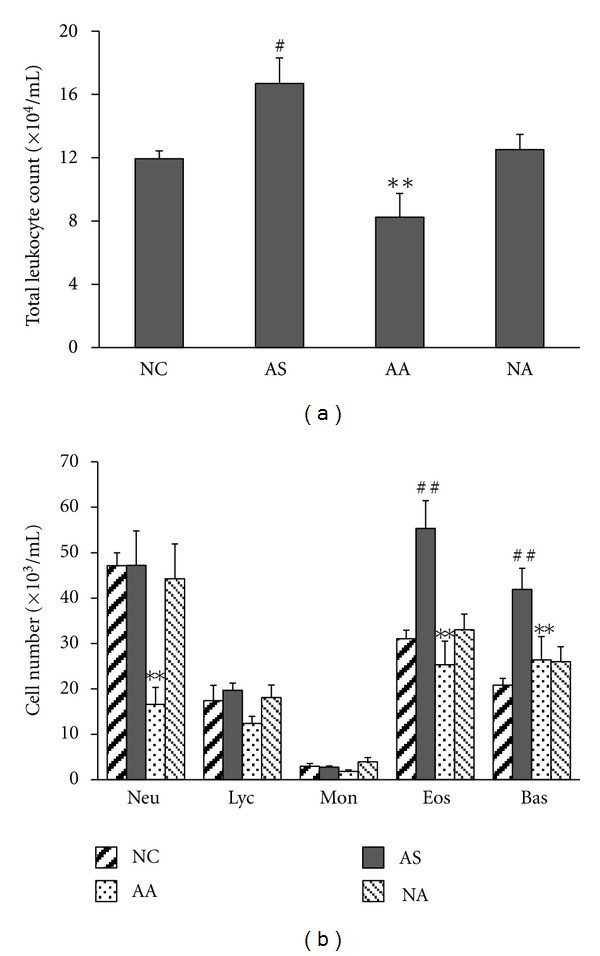
The effect of acupuncture on the cellular composition of BALF. BALFs were collected after OVA challenge. Total leukocyte (a) and differential cell counts (b) were performed. Neu, neutrophil; Lym, lymphocyte; Mon, monocyte; Eos, eosinophil; Bas, basophil. Values represent the mean ± SEM. ^#^ *P* < 0.05, ^##^ *P* < 0.01 when comparing the AS group with the NC group, and **P < 0.01 when comparing the AA group with the AS group.

The total leukocyte, eosinophil, and basophil counts in BALF recovered from OVA-challenged rats were significantly higher compared to normal control rats (*P* < 0.05, *P* < 0.01, and *P* < 0.01, resp.). However, the numbers of neutrophils, lymphocytes, and monocytes were unchanged after OVA challenge in OVA-sensitized rats. Treatment with acupuncture in the AA group significantly decreased the total leukocyte, neutrophils, eosinophils, and basophils in BALF compared with AS rats (all *P* < 0.01). The BALF total and differential cell counts were not significantly different between the normal control rats and acupuncture-treated normal rats (Figures 3(a) and 3(b)), which indicated that the changes in the cellular composition of the BALF by acupuncture is specific to asthma onset.

### 3.3. Changes in the Lung Proteome of Asthma Onset in Rats Treated with Acupuncture

**Figure 4:**
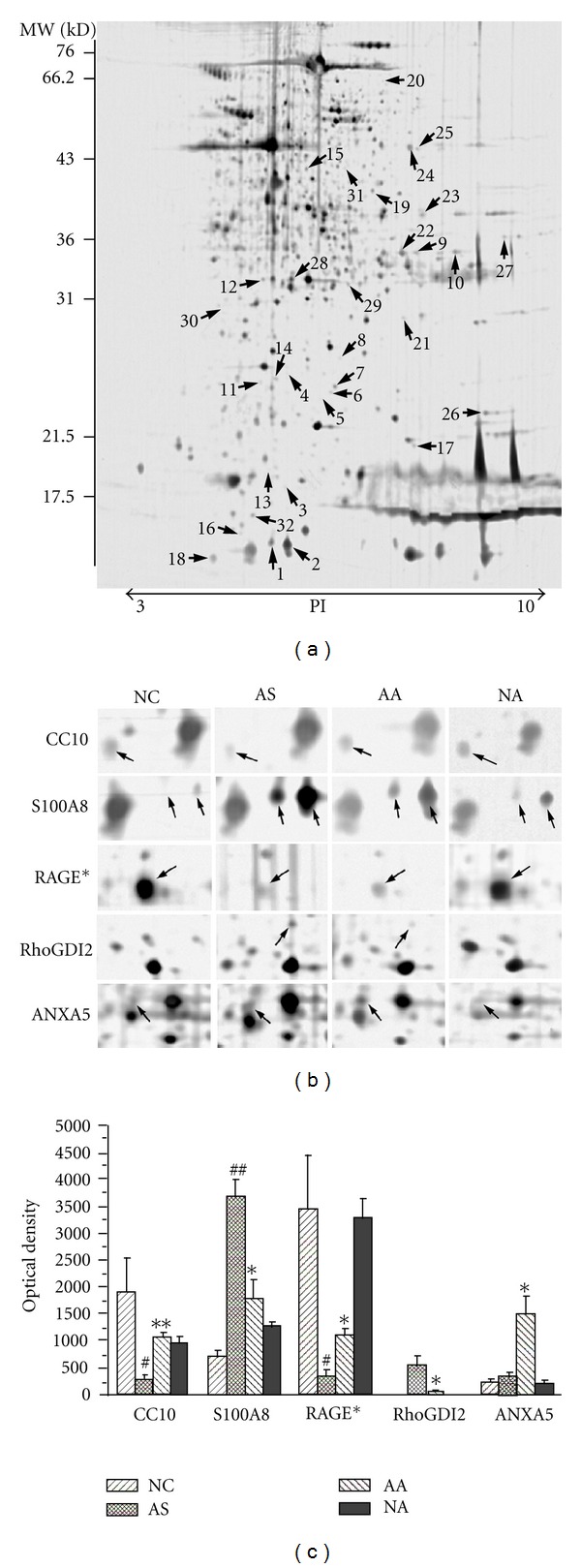
Protein expression profiles in lung tissues. (a) A representative 2DE gel image of proteins isolated from the AA group. The numerically labeled spots indicate the differentially expressed protein spots among the NC, AS, and AA groups. The numbers correspond to the spot identification numbers listed in Table 1. The molecular weight standards and pH range are shown at the left and bottom of the gels, respectively. (b) Differential expression profiles of five inflammation-related proteins regulated by acupuncture: CC10, S100A8, RAGE* (which is actually sRAGE, Table 1), RhoGDI2, and ANXA5. The cropped images of 2DE gels were symmetrically boxed, and the arrows on the images indicate the relative positions of the protein spots. (c) Quantitative analysis of the five inflammation-related proteins regulated by acupuncture. Each spot volume was quantified from the intensity of the spots using PDQuest software. The bars represent the mean ± SEM of triplicate 2DE gels. ^#^ *P* < 0.05, ^##^ *P* < 0.01 when comparing the AS group with the NC group. *P < 0.05, **P < 0.01 when comparing the AA group with the AS group.

The protein content in the sample from each group was measured using 2DE at least three times and was followed up by verifying that the same protein patterns within each group were obtained. We detected approximately 550–650 distinct protein spots in each 2DE gel and acquired a high overlap rate (>85%) for spots in the parallel gels. Image analysis of the 2DE gels revealed that acupuncture at specific points induced lung proteome changes both in the normal rats (NA group versus NC group) and in rats with asthma onset (AA group versus AS group). However, the expression levels of 32 protein spots were altered after acupuncture treatment in the rats with asthma onset but not changed after acupuncture treatment in the normal rats (Figure 4(a)), which essentially indicated the pathology-specific regulation of acupuncture. These 32 acupuncture-specific differentially expressed protein spots could be divided according to their 2DE gel expression patterns into six different categories, which are presented in Table 1. Thirty protein spots were successfully identified by LC-MS/MS, which corresponded to 28 unique proteins (Table 1). Among them, 23 proteins had differential expression levels in the AS group compared with the NC group, and the levels were restored to normal expression levels after acupuncture in the AA group, including the proteins CC10 (uteroglobin), S100A8, RAGE (advanced glycosylation end product-specific receptor), and RhoGDI2 (Rho, GDP dissociation inhibitor (GDI) beta) (Figures 4(b), and 4(c)). ANXA5 (Annexin A5) and PRDX6 (Peroxiredoxin 6) did not exhibit changes in expression in the AS group compared with the NC group; however, they did exhibit increased and decreased levels, respectively, after acupuncture in the AA group. 

### 3.4. Bioinformatic Analyses of Proteins Differentially Expressed following Acupuncture

**Figure 5:**
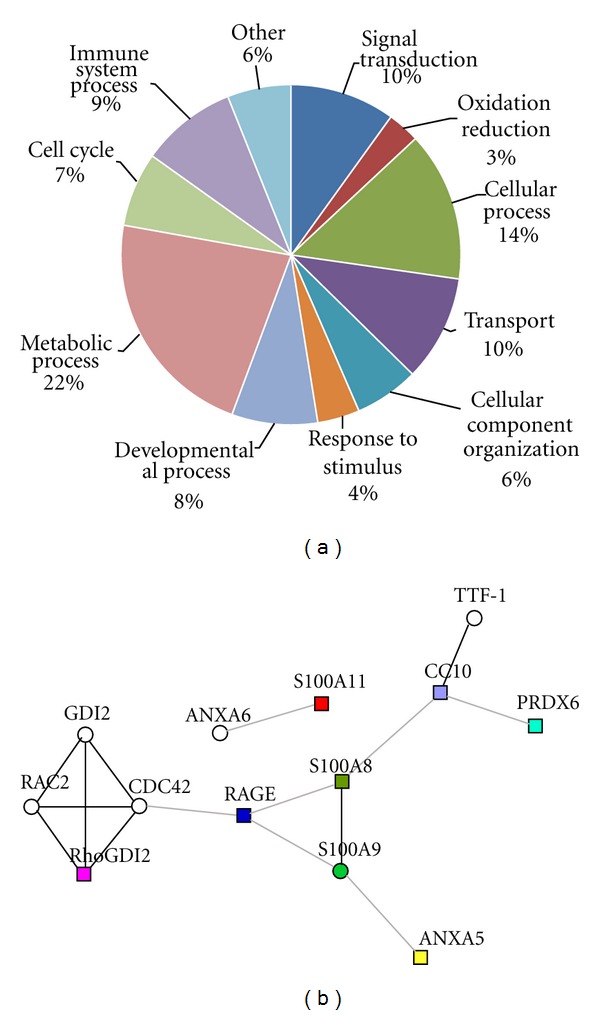
Bioinformatic analysis of the acupuncture-specific differentially expressed proteins in asthma onset. (a) Functional classification of the identified proteins using the PANTHER classification system. Proteins that were differentially expressed following acupuncture were classified into diverse functional categories according to biological process. (b) The STRING network of known protein-protein interactions among the seven inflammation-related proteins (marked by colored rectangles) regulated by acupuncture. Circle nodes indicate intermediate proteins not detected in the proteomic study (RAC2, RAS-related C3 botulinum substrate 2; GDI2, Rab GDI beta; CDC42, Cell division control protein 42 homolog; ANXA6, Annexin A6; TTF-1, Thyroid transcription factor 1). S100A9 (green circle) is an inflammation-related protein regulated by acupuncture, which was determined in our previous study [16]. The network edges represent the predicted functional associations, and stronger associations are represented by darker lines.

Using the PANTHER classification system, proteins that were differentially expressed following acupuncture in the AA group were classified by biological processes (Figure 5(a), Table Supplemental 2 available online at doi:10.1155/2012/850512). Protein classification revealed that many of the identified proteins are implicated in inflammation-related biological functions, including metabolic processes, inflammatory signal transduction, immune system processes, response to stimulus, and oxidation/reduction. The effect of Acupuncture on asthma may be associated with the regulation of inflammatory processes.

Further analysis with the online tool STRING revealed a protein-protein interaction network among the proteins with inflammation-related biological functions, and seven proteins, including CC10, S100A8, S100A11, RAGE, ANXA5, RhoGDI2, and PRDX6, were associated either directly or indirectly by intermediate proteins (proteins that have known interactions with the identified proteins but were not detected in our proteomic experiment) (Figure 5(b)). Notably, the intermediate protein S100A9, which is an inflammation-related protein related to S100A8 in terms of biological function, was regulated in asthmatic rats treated with acupuncture in our previous study [16]. The functional associations of these proteins indicate an inflammatory signaling pathway in asthma that can be regulated by acupuncture. 

### 3.5. Confirmation of the Differential Expression of Inflammation-Related Proteins

**Figure 6:**
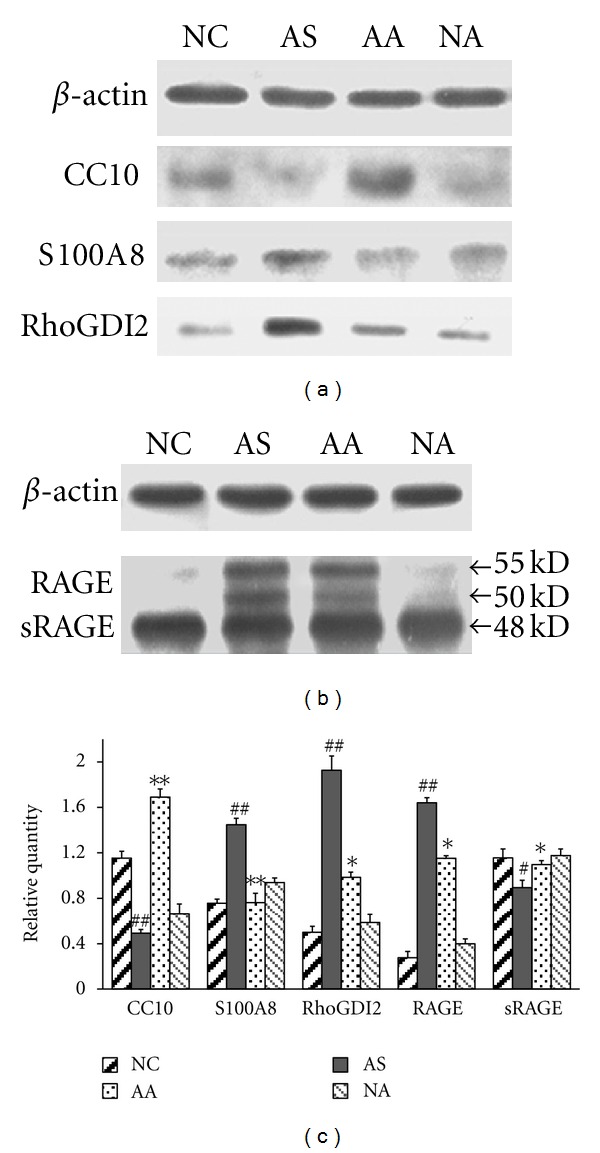
Validation of the expression profiles of five inflammation-related proteins by western blot analysis. (a) A representative western blot visualizing the expression levels of CC10, S100A8, and RhoGDI2. *β*-actin was used to demonstrate equal loading. (a) Western blot analysis using the anti-rat RAGE extracellular domain monoclonal goat antibody demonstrates that the lung homogenate contains three bands that were approximately 48, 50, and 55 kD in size; the 48-kD band corresponds to sRAGE and the 55 and 50 kD bands correspond to full-length RAGE. (b) The densitometric quantification of individual proteins is expressed as the fold change compared to *β*-actin. Each bar represents the mean ± SEM of triplicate experiments, and similar results were observed in all experiments. ^#^ *P* < 0.05, ^##^ *P* < 0.01 when comparing the AS group with the NC group; *P < 0.05, **P < 0.01 when comparing the AA group with the AS group.

To verify the deregulation of inflammation-related proteins following acupuncture, CC10, S100A8, RAGE, and RhoGDI2 were selected as representative proteins and subjected to western blotting. In the AS group, the expression of S100A8 and RhoGDI2 were significantly increased (*P* < 0.01), whereas the expression of CC10 was significantly decreased (*P* < 0.01) when compared with the NC group. In the AA group, the expression of S100A8 and RhoGDI2 was downregulated (*P* < 0.01 and *P* < 0.05, resp.), and the expression of CC10 was upregulated (*P* < 0.01) relative to the AS group after acupuncture (Figures 6(a) and 6(c)). These results are consistent with the results observed in the 2DE gels. 

RAGE typically consists of an extracellular immunoglobulin-like region, a short transmembrane domain, and a cytosolic tail. In addition to full-length RAGE, there is an isoform designated as soluble RAGE (sRAGE), which corresponds to the extracellular domain of RAGE lacking the cytosolic and transmembrane domains [20]. AS to differentially expressed protein spot 24 (Figure 4), all six unique peptide sequences identified by mass spectrometry were mapped to the extracellular domain of RAGE (Table Supplemental 3 available online at doi:10.1155/2012/850512). Therefore, it is possible that the identified protein corresponding to spot 24 was sRAGE as opposed to RAGE. To investigate this possibility, we performed western blot experiment using an anti-rat RAGE extracellular domain monoclonal goat antibody (R&D Systems; Minneapolis, MN), which demonstrated that the lung tissue homogenate contained three protein bands of approximately 55, 50, and 48 kD; the 48 kD band corresponds to sRAGE and the 55 kD and 50 kD bands correspond to full-length RAGE as described by Uchida et al. [21]. The resulting expression pattern is consistent with the 2DE analysis that shows that sRAGE was expressed at a relatively low level in the AS group (*P* < 0.05 versus NC) and was upregulated after acupuncture in the AA group (*P* < 0.05 versus AS). However, the expression pattern of RAGE is completely opposite to that of sRAGE (Figures 6(b) and 6(c)).

## 4. Discussion 

Acupuncture is an integral part of traditional Chinese medicine and is used to treat variety of illnesses, including asthma. Over the past decade, several clinical trials have evaluated the efficacy of acupuncture therapy on asthma [22, 23]. In our recent clinical research, we have reported that acupuncture at the *Dazhui, Fengmen, *and* Feishu* points of patients with allergic asthma has an immunomodulatory effect, which results in an improvement in general well-being [8]. In view of the theory of traditional Chinese medicine, *Dazhui*, which is the crossing point of three *yang* meridians and the governor vessel (GV), can strengthen the* yang qi *of the entire body and reinforce the *vital qi *to relieve asthmatic symptoms. *Fengmen* always functions to regulate the flow of the meridian* qi* and is used to dredge and activate the lung meridian. *Feishu *is the site where the *qi* of the lung meridian gathers in the back and is especially effective in treating respiratory disorders caused by either exogenous or endogenous pathogenic factors. The combination of the three points can regulate the *qi* of the lung meridian, remove obstruction from the meridian, and disperse the lung *qi* to stop asthma.

Although the antiasthmatic effect of acupuncture has been well documented, little is known about its molecular basis. In the present study, acupuncture treatment appeared to improve the respiratory function and reduce the total leukocyte and eosinophil number in the bronchoalveolar lavage fluid of sensitized rats after OVA challenge. Using a 2DE/MS-based proteomic approach, the lung proteome of acupuncture-treated rats with asthma onset was examined. Acupuncture at specific points led to lung proteome changes both in the normal rats (NA group versus NC group) and the rats with asthma onset (AA group versus AS group). However, the expression levels of 32 protein spots were observed to change after acupuncture treatment in rats with asthma onset but were unchanged after acupuncture treatment in normal rats. 30 of the 32 acupuncture-specific differentially expressed protein spots in asthma onset were successfully identified as 28 unique proteins. The identified proteins have been implicated in diverse inflammation-related biological functions, including inflammatory signal transduction, immune system processes, response to stimulus, and oxidation/reduction. Of these proteins, several proinflammatory proteins (e.g., S100A8, RAGE, and S100A11) were downregulated, and several anti-inflammatory proteins (e.g., CC10, ANXA5, and sRAGE) were upregulated following acupuncture. The investigation of protein-protein interactions revealed a functional network associated with inflammatory signaling, and CC10, S100A8, RAGE, and RhoGDI2 acted as key node proteins. Western blot analysis of CC10, S100A8, RAGE, and RhoGDI2 confirmed the regulation of inflammation-related proteins and the functional network by acupuncture treatment.

S100 proteins, which are characterized by two EF-hand calcium-binding motifs, have received increased attention in the study of asthma because of their close association with inflammation [24, 25]. We previously reported that the increased mRNA expression of S100A9 (also known as calgranulin B; MRP14), a calcium-binding protein in the S100 protein family, was downregulated to control levels after acupuncture [16]. In the current study, a similar regulation of the expression of two S100 proteins, S100A8 (also known as calgranulin A, MRP8) and S100A11 (also known as calgizzarin, S100C), was observed in acupuncture-treated OVA-induced asthma onset rats. S100A8 and S100A9 are small calcium-binding proteins that are highly expressed in neutrophil and monocyte cytosols and generally form a heterodimer (S100A8/A9, calprotectin) in a calcium-dependent manner [25]. Increasing evidence indicates that S100A8 and S100A9 are involved in intracellular and extracellular regulatory activities. Within cells, S100A8 and S100A9 play important roles in regulating enzyme activities, cytoskeleton dynamics, transcription factors, Ca^2+^ homeostasis, and the inflammatory response [26]. In addition, S100A8 and S100A9 are secreted into the extracellular space by unknown mechanisms [26, 27]. Secreted S100A8 and S100A9 proteins can act in a cytokine-like manner as extracellular ligands for cell surface receptors, including RAGE, which activates signaling cascades and initiates cellular responses [27]. Furthermore, S100A8 and S100A9 are involved in a novel proinflammatory signaling pathway where they contribute to the activation of central cellular pathways, including p38 or p44/42 MAP kinases and NF-*κ*B signaling components, which induce the expression of many proinflammatory molecules [27, 28]. Elevated levels of S100A8 and S100A9 in human asthmatic BALF are strongly linked to allergic inflammation [29]. Moreover, the inhibition of S100A8 and S100A9 reduced the migration of inflammatory cells into the lungs in a mouse model of asthma [30]. These observations suggest an association between pulmonary inflammation and S100A8 and S100A9. In our studies, the increased expression levels of S100A8, S100A9, and S100A11 in asthma onset in rats were downregulated after acupuncture, which suggests that acupuncture had an effect on the regulation of inflammatory reactions in asthma.

RAGE is a member of the immunoglobulin superfamily of cell surface receptors. RAGE binds to multiple ligands, including S100 proteins, which leads to the activation of proinflammatory signaling pathways, such as Cdc42/Rac, MAPK (ERK, p38, and JNK) [27], PI3 K, and Rho/Rho-kinase [31], as well as the up-regulation of RAGE itself. Although RAGE binds many S100 proteins, such as S100B, S100A1, and S100A12, it is unknown if RAGE directly binds to S100A8, S100A9, or S100A8/A9 heterodimers [32]. Recent studies have provided evidence that these interactions are likely [27, 33, 34] and may explain the similarities between the expression patterns of RAGE, S100A8 and S100A9 in our study, which were increased in the AS group relative to the NC group and were downregulated to control levels after acupuncture in the AA group. Notably, acupuncture simultaneously restored the expression level of sRAGE, a secretory isoform of RAGE that was expressed at a relatively low level in the AS group. sRAGE lacks the transmembrane domain found in intact RAGE and acts as an endogenous inhibitor of RAGE by binding circulating ligands and inhibiting RAGE-induced proinflammatory signaling, tissue damage, and dysfunction [20]. Treatment of animals with sRAGE can prevent and even reverse RAGE-mediated pathological processes [31, 35]. Acupuncture could inhibit the S100 protein/RAGE-mediated inflammatory signaling pathway by downregulating the proteins S100A8, S100A9, and RAGE while upregulating sRAGE expression, which would to reverse the inflammatory process of asthma.

Protein CC10 (also known as uteroglobin, and secretoglobin 1A member 1) is a steroid-inducible, evolutionarily conserved, secreted protein of the secretoglobin family [36]. CC10 is mainly produced from Clara cells, which are nonciliated cells located mainly in the bronchioles, and functions as an important anti-inflammatory mediator in the lung [37]. There is a marked decrease in the synthesis and secretion of CC10 during LPS-induced acute lung inflammation [38]. Increased levels of IL-4, IL-5, and IL-13 [39] as well as a severe inflammatory response [40] have been observed in the lungs of CC10-deficient mice after allergen challenge, which confirms the functional relevance of CC10 in allergic inflammation. Treatment with CC10 could improve airway conditions where inflammation is part of the pathophysiology [41, 42]. CC10 plays a key role as an important endogenous regulator that modulates pulmonary inflammatory reactions. In the present study, acupuncture upregulated CC10 expression, which was decreased in OVA-sensitized/challenged rats relative to normal control rats and indicates an anti-inflammatory effect of acupuncture on asthma. In agreement with the reported functional associations among CC10, S100A8, S100A9, and RAGE in CC10-knockout mice [43], our study showed that the asthma onset in rats with decreased expression of CC10 overexpressed two calcium-binding proteins, S100A8 and S100A9, which were coupled with increased RAGE expression. These results suggest an inflammatory pathway in asthma consisting of CC10, RAGE, S100A8, and S100A9. Acupuncture in asthma appears to exert an anti-inflammatory effect by regulating these inflammation-related proteins and inhibits this pathway.

Using the PANTHER classification system, seven of the identified proteins (Table Supplemental 2 available online at doi:10.1155/2012/850512) were classified as being involved in signal transduction processes, which include cell-cell signaling, S100 protein/RAGE signaling, and Rho (Rho GTPase proteins)/Rho-kinase signaling. The Rho/Rho-kinase-mediated signaling pathway plays a crucial role in several pathophysiological processes of asthma, including airway smooth muscle contraction, airway remodeling, and airway inflammation [44]. Inactivation of Rho/Rho-kinase signaling could inhibit the infiltration of airways by inflammatory cells and airway hyperresponsiveness induced by allergen challenge [45]. Several compounds, such as Y-27632 and fasudil, have been developed to prevent or block the inflammatory response in asthma by inhibiting the Rho/Rho-kinase signaling pathway [44, 46]. RhoGDI2 plays a role in the regulation of Rho/Rho-kinase signaling by negatively regulating the biological function of Rho GTPases [47]. In contrast, RhoGDIs may act as positive regulators required for the correct targeting and regulation of Rho GTPases [48]. In the asthmatic lung proteome, significantly increased RhoGDI2 expression was downregulated after acupuncture, indicating an association between Rho/Rho-kinase signaling and the antiasthma effect of acupuncture; however, the precise role of RhoGDI2 in the Rho/Rho-kinase-mediated signaling pathway is not clear. Further studies of RhoGDI2 and the effects on Rho/Rho-kinase signaling will determine if RhoGDI2 is a novel therapeutic target for asthma.

We also detected decreased expression of PRDX6 and increased expression of ANXA5 in the acupuncture-treated model; however, these two proteins did not exhibit altered expression in asthma onset in rats when compared with the controls. ANXA5 is a member of the annexins superfamily of calcium- and phospholipid-binding, which have been linked to fibrinolysis, coagulation, apoptosis, and inflammation [49]. The anti-inflammatory properties of ANXA5 affect the functional consequences of inflammation in the vessel wall [50]. PRDX6 is a novel antioxidant enzyme that is highly expressed in the lung and plays an important role in the defense against oxidative lung damage [51]. Deregulations of ANXA5 and PRDX6 suggest a regulatory effect of acupuncture on pulmonary inflammation and oxidative stress.

**Figure 7:**
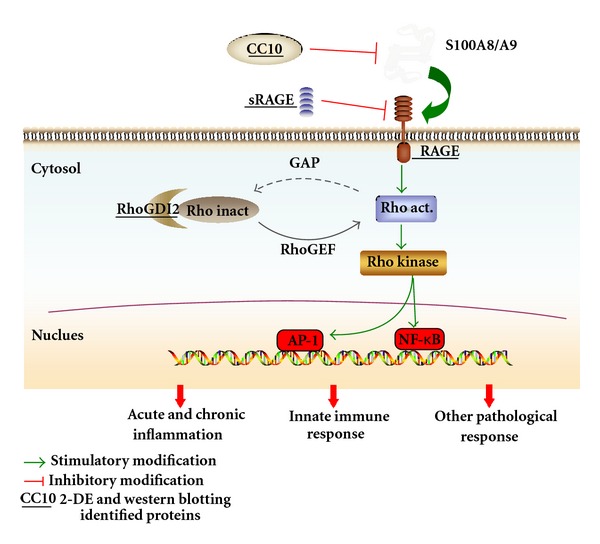
A possible inflammatory signaling pathway that results from the functional associations of the identified inflammation-related proteins (marked with an underline), which is regulated by acupuncture in asthma. Acupuncture can downregulate the proteins S100A8, S100A9, RAGE, and RhoGDI2 and upregulate the expression of CC10 and sRAGE. This pathway may explain the anti-inflammatory effect of acupuncture in asthma. Rho inact, inactivated Rho GTPase; Rho act, activated Rho GTPase; RhoGEF, Rho-specific guanine nucleotide exchange factors; GAP, GTPase-activating proteins; NF-*κ*B, nuclear factor-*κ*B; AP1, activator protein-1.

Airway inflammation is the dominant pathophysiological process in the natural course of asthma [1]. We previously reported a proinflammatory process during the early asthmatic response, and calcium binding played a central role in this process [18]. In this study, several inflammation-related proteins were regulated in the lungs of rats treated with acupuncture, including downregulated proinflammatory proteins and upregulated anti-inflammatory proteins. The functional association of these deregulated proteins suggests an inflammatory signaling pathway that might play a crucial role in the inflammatory reactions of asthma (Figure 7). In this pathway, the low level of CC10 likely stimulates the production of S100A8 and S100A9, which activates the S100 protein/RAGE-mediated proinflammatory signaling pathway in the lungs of asthmatic rats. The binding of RAGE to its S100 ligands provides a channel for the transduction of inflammatory signals into the cell, which further activates the intracellular Rho/Rho-kinase signaling pathway mediates the activation and nuclear translocation of transcription factors, such as nuclear factor (NF)-*κ*B and activator protein (AP)-1, and eventually leads to the inception of the early airway inflammatory process. Acupuncture can systematically regulate this inflammatory signaling pathway at different levels through the regulation of several key nodal proteins, including CC10, S100A8, S100A9, RAGE, sRAGE, and RhoGDI2 (Figure 7), which may explain, at least in part, the anti-inflammatory effect of acupuncture in asthma.

In summary, this paper is the first to report the use of a proteomic approach to analyze the protein profile of an asthma model treated with acupuncture. Our results indicate that acupuncture downregulates the expression of proinflammatory proteins (e.g., S100A8, RAGE, and S100A11) and upregulates the expression of anti-inflammatory proteins (e.g., CC10, ANXA5, and sRAGE) in the lung tissues of rats with asthma onset. Characterizing the balance and interaction between these inflammation-related proteins may contribute to a better understanding of the molecular mechanisms that underlie the antiasthmatic effect of acupuncture. Moreover, further functional investigations of these acupuncture-specific effector proteins could identify new drug candidates for the prophylaxis and treatment of asthma.

## Supplementary Material

Figure 1. Rats on the suspended shelf. For the convenient manipulation of the acupuncture points on the back, rat was placed on the suspended shelf (50 × 45 mm, about 50 cm high from the ground, shown as below), which could easily make it calm and stand still without anesthesia.
Table 1. The comparisons of pulmonary dynamic compliance (Cdyn, ml/kpa) and respiratory rate (RR, breaths/min) within 10 min after challenge.
Table 2. List of PANTHER biologic process classification.
Table 3 (a). Mass spectrometric data of differentially expressed protein spot 24.
Table 3 (b). Peptide sequences matched to RAGE (residues 1–342 correspond to the extracellular domain∗) are shown below.

## Figures and Tables

**Table 1:** List of 32 acupuncture-specific differentially expressed proteins in asthma onset.

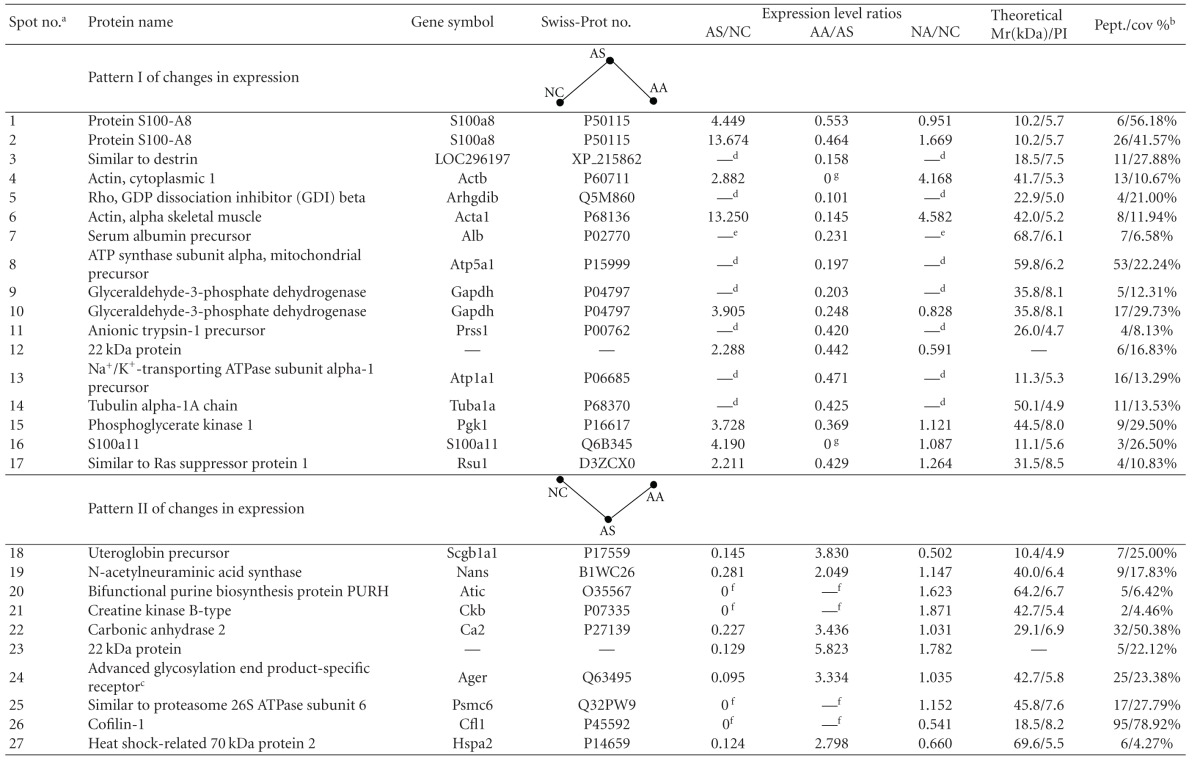 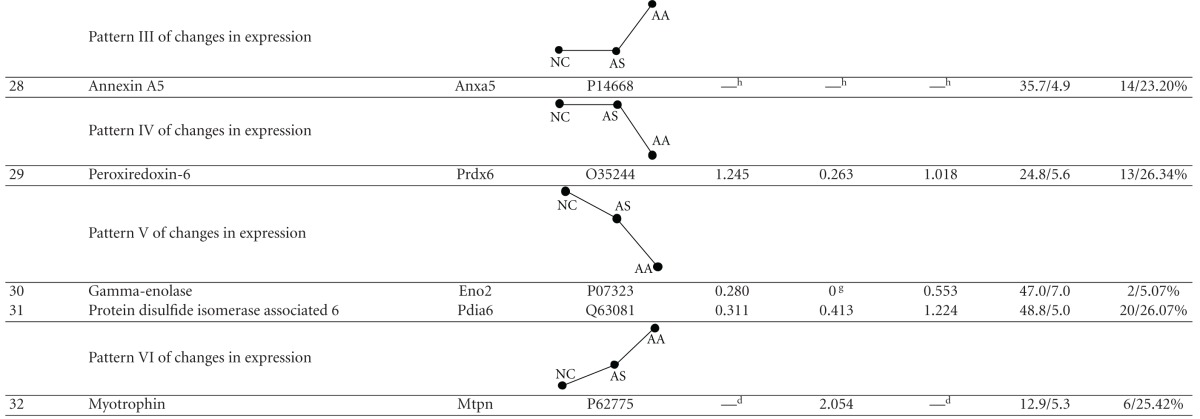

The 32 acupuncture-specific differentially expressed protein spots in asthma onset can be divided into six different categories according to their expression patterns on 2DE gels (NC: normal control, AS: asthma model, AA: asthma model treated with acupuncture, and NA: normal rats treated with acupuncture). Patterns I and II, proteins that exhibited either increased or decreased levels in AS compared with NC that were restored to normal expression levels by acupuncture; Patterns III and IV, proteins that did not exhibit changed levels in AS compared with NC but did exhibit either increased or decreased levels after acupuncture; Patterns V and VI, proteins that exhibited either increased or decreased levels in AS compared with NC and exhibited similar expression patterns after acupuncture in AA compared with NC.

^a^The spot number from 2DE gel analysis (Figure 3); ^b^the number of matching peptides (Pept.) and percentage of the total amino acid sequence covered by the peptides (% Seq.Cov) from the mass mapping experiments; ^c^protein spot 24 is actually the soluble receptor for advanced glycation end products (sRAGE), which corresponds to the extracellular domain of full-length RAGE lacking the cytosolic and transmembrane domains, as revealed by BLAST and verified by western blot analysis; ^d^spot not detected in gels from the NC and NA samples; ^e^spot not detected in gels from the NC samples; ^f^spot not detected in gels from the AS samples; ^g^spot not detected in gels from the AA samples; ^h^spot detected only in the AA samples.
