# Supplementary material for: Proteomic Analysis Reveals the Deregulation of Inflammation-Related Proteins in Acupuncture-Treated Rats with Asthma Onset
Source: Evid Based Complement Alternat Med. 2012 Nov 14;2012:850512. doi: 10.1155/2012/850512 (PMC3523810; doi:10.1155/2012/850512)
Supplement: Supplementary file 1 — Figure 1. Rats on the suspended shelf. For the convenient manipulation of the acupuncture points on the back, rat was placed on the suspended shelf (50 × 45 mm, about 50 cm high from the ground, shown as below), which could easily make it calm and stand still without anesthesia. Table 1. The comparisons of pulmonary dynamic compliance (Cdyn, ml/kpa) and respiratory rate (RR, breaths/min) within 10 min after challenge. Table 2. List of PANTHER biologic process classification. Table 3 (a). Mass spectrometric data of differentially expressed protein spot 24. Table 3 (b). Peptide sequences matched to RAGE (residues 1–342 correspond to the extracellular domain∗) are shown below. [file 850512.f1.pdf]

**Supplemental Figure 1.** Rats on the suspended shelf. For the convenient manipulation of the acupuncture points on the back, rat was placed on the suspended shelf (50 × 45 mm, about 50 cm high from the ground, shown as below), which could easily make it calm and stand still without anesthesia.

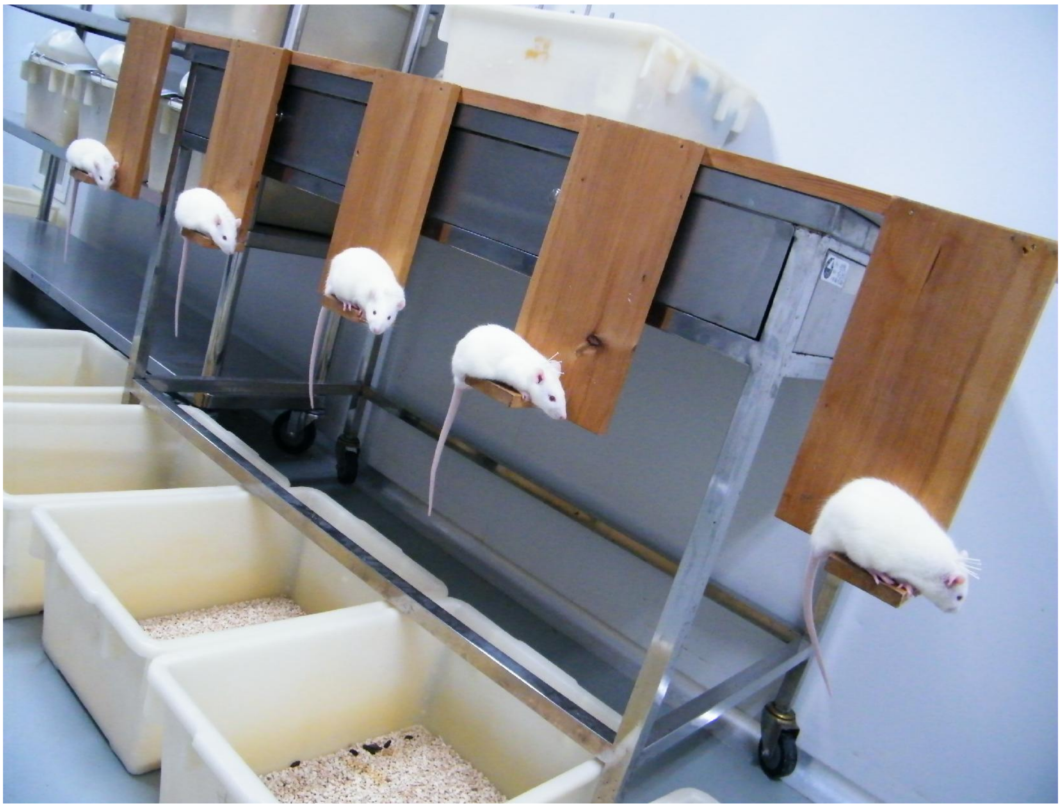

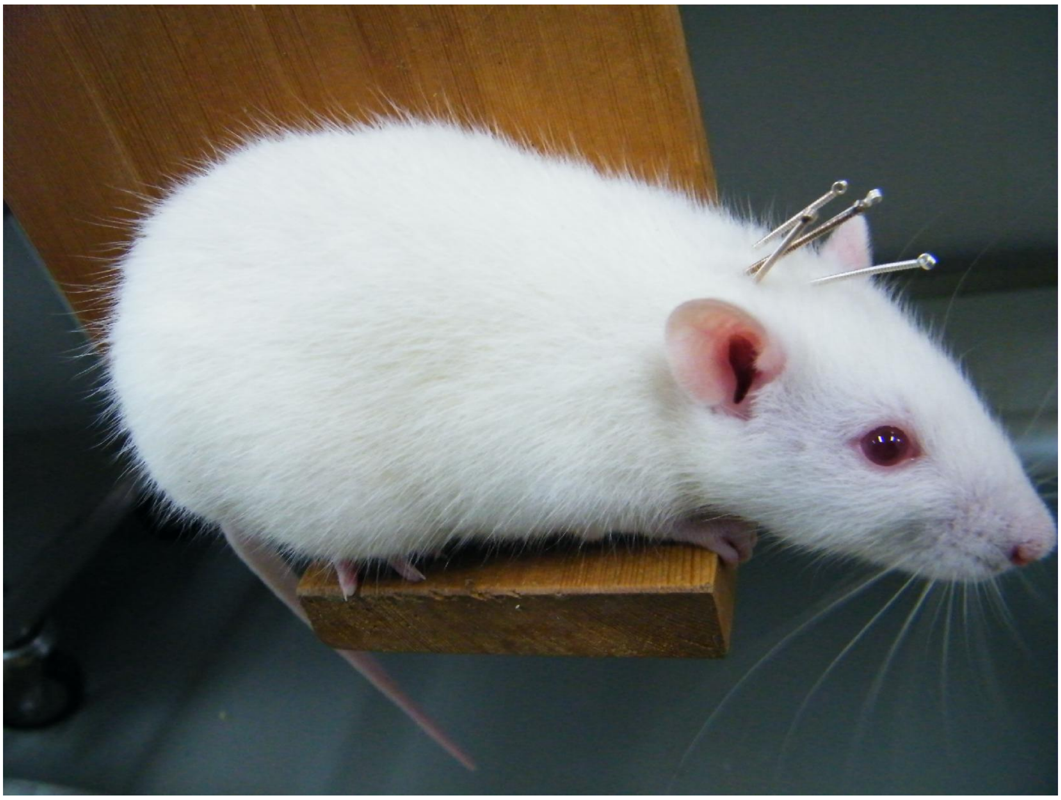

**Supplemental Table 1. The comparisons of pulmonary dynamic compliance (Cdyn, ml/ kpa) and respiratory rate (RR, breaths/min) within 10min after challenge**

| | Groups | Min 1 | Min 2 | Min 3 | Min 4 | Min 5 | Min 6 | Min 7 | Min 8 | Min 9 | Min 10 |
|---|---|---|---|---|---|---|---|---|---|---|---|
| **Cdyn** | NC | 1.066±2.796 | 0.463±2.609 | -0.373±2.496 | -2.128±2.625 | -1.53±2.836 | -2.25±3.179 | -2.208±3.117 | -4.015±2.542 | -5.605±2.933 | -3.679±3.747 |
| | AS | -3.629±3.493 | -20.434±5.057## | -23.641±5.242## | -17.95±6.017# | -12.561±5.277# | -3.274±5.035 | 3.584±5.266 | 6.292±5.018# | 5.992±4.642# | 5.698±4.240 |
| | AA | -0.319±3.512 | 3.198±5.929** | 7.435±7.046** | 3.751±6.623** | 4.322±5.367** | 3.725±5.11 | 8.03±5.638 | 7.889±4.385 | 6.86±3.744 | 5.707±3.458 |
| | NA | 1.019±2.736 | 0.417±1.238 | -1.095±1.338 | -0.14±1.154 | -0.959±1.12 | -1.923±1.562 | -0.883±1.109 | -0.552±1.232 | -1.503±1.303 | -2.071±1.656 |
| **RR** | NC | 7.04±2.496 | 2.084±1.233 | 1.5±1.308 | 1.871±1.301 | 1.974±1.419 | 3.238±1.439 | 3.934±1.365 | 3.898±1.434 | 4.352±1.734 | 5.465±2.433 |
| | AS | -28.948±5.912## | -53.031±8.193## | -46.9±6.911## | -39.065±5.768## | -28.048±2.843## | -17.874±3.708## | -13.32±5.078## | -13.033±4.697## | -13.178±4.731## | -12.583±5.065## |
| | AA | -29.822±8.505 | -48.332±11.297 | -36.24±9.328 | -25.474±8.025 | -21.728±7.631 | -18.959±6.143 | -15.501±4.685 | -15.508±4.221 | -14.934±4.449 | -17.906±5.674 |
| | NA | 4.055±1.366 | 0.506±1.162 | 1.465±0.927 | 2.251±1.182 | 2.536±1.143 | 3.521±1.387 | 3.094±1.203 | 3.287±1.336 | 4.173±1.84 | 6.662±2.225 |

Data are shown as means ± SEM. NC: normal control rats (n = 15); AS: asthma model (n = 14); AA: asthma model treated with acupuncture (n = 12); NA: normal rats treated with acupuncture. The values of Cdyn and RR in the tables are expressed as differential values subtracted from the corresponding baseline values (Figure 1). # $p < 0.05$, ## $p < 0.01$ when comparing the AS group with the NC group and * $p < 0.05$, ** $p < 0.01$ when comparing the AA group with the AS group, , one-way ANOVA followed by LSD post hoc comparisons.

**Supplemental Table 2. List of PANTHER biologic process classification**

| Biological process | Number of process hits | Identified proteins |
|---|---|---|
| signal transduction | 7 | RhoGDI2, S100A8, S100A11, RAGE, Uteroglobin, Annexin A5, Ras suppressor protein 1 |
| oxidation reduction | 2 | Peroxiredoxin-6, Protein disulfide-isomerase A6 |
| cellular process | 10 | RhoGDI2, S100A8, S100A11, RAGE, Uteroglobin, Annexin A5, Tubulin alpha-1A chain, Actin, cytoplasmic 1, Actin, alpha skeletal muscle, Cofilin-1 |
| transport | 7 | Serum albumin, ATP synthase subunit alpha, mitochondrial, Sodium/potassium-transporting ATPase subunit alpha-1, Tubulin alpha-1A chain, Annexin A5, Actin, cytoplasmic 1 |
| cellular component organization | 4 | Tubulin alpha-1A chain, Actin, cytoplasmic 1, Actin, alpha skeletal muscle, Cofilin-1 |
| response to stimulus | 3 | S100A8, S100A11, Heat shock-related 70 |
| developmental process | 6 | RAGE, N(G),N(G)-dimethylarginine dimethylaminohydrolase 2, Tubulin alpha-1A chain, Actin, cytoplasmic 1, Actin, alpha skeletal muscle, Cofilin-1 |
| metabolic process | 16 | Peroxiredoxin-6; Protein disulfide-isomerase A6; Atic; Psmc6; Glyceraldehyde-3-phosphate dehydrogenase; Anionic trypsin-1; Gamma-enolase; Phosphoglycerate mutase 1; Carbonic anhydrase 2; synthase subunit alpha, mitochondrial; Sodium/potassium-trtransporting ATPase subunit alpha-1 |
| cell cycle | 5 | S100A8, S100A11, Tubulin alpha-1A chain, Actin, cytoplasmic 1, Actin, alpha skeletal muscle |
| immune system process | 6 | Peroxiredoxin-6, S100A8, S100A11, uteroglobin, RAGE, Heat shock-related 70 kDa protein 2 |
| other | 4 | RAGE, Sodium/potassium-transporting ATPase subunit alpha-1, ATP synthase subunit alpha, mitochondrial, Creatine kinase B-type |

The number of process hits is greater than the number of proteins since many proteins are classified as having multiple functions. ‘‘Other’’ category contains biological processes with only 1 hit: homeostatic process, cell adhesion, system process, generation of precursor metabolites and energy.

## Supplemental Table 3 (a). Mass spectrometric data of differentially expressed protein spot 24

| **Reference** IPI:IPI00209188.5\|SWISS-PROT:Q63495\|TREMBL:Q6MG86\|ENSEMBL:ENSRNOP00000000508\|REFSEQ:NP_445788 | | | | | | | |
|---|---|---|---|---|---|---|---|
| **Advanced glycosylation end product-specific receptor precursor** | | | PepCount | UniquePepCount | CoverPercent | MW | PI |
| | | | 25 | 6 | 23.38% | 42663.67 | 5.83 |
| File, Scan(s) | Sequence | MH+ | Diff(MH+) | Charge | Rank | XC | DeltaCn |
| 7503_Test,1570 | K.GTVVKEETR.R | 1019.1343 | 0.1823 | 2 | 1 | 2.4614 | 0.353 |
| 7503_Test,1576 | K.GTVVKEETR.R | 1019.1343 | 0.1303 | 2 | 1 | 2.8472 | 0.389 |
| 7503_Test,1588 | K.GTVVKEETR.R | 1019.1343 | 0.5963 | 2 | 1 | 2.7144 | 0.4287 |
| 7503_Test,1860 | K.GTVVKEETR.R | 1019.1343 | 0.1753 | 2 | 1 | 2.9238 | 0.3669 |
| 7503_Test,1872 | K.GTVVKEETR.R | 1019.1343 | 0.1403 | 2 | 1 | 2.7705 | 0.423 |
| 7503_Test,1878 | K.GTVVKEETR.R | 1019.1343 | 0.1893 | 2 | 1 | 2.7504 | 0.3201 |
| 7503_Test,3081 | K.VLSPQGDPWDSVAR.I | 1527.66322 | -0.80178 | 2 | 1 | 4.065 | 0.6215 |
| 7503_Test,3087 | K.VLSPQGDPWDSVAR.I | 1527.66322 | 0.95522 | 2 | 1 | 3.9653 | 0.6064 |
| 7503_Test,3093 | K.VLSPQGDPWDSVAR.I | 1527.66322 | 0.24722 | 2 | 1 | 4.0732 | 0.6465 |
| 7503_Test,3369 | K.VLSPQGDPWDSVAR.I | 1527.66322 | 0.99522 | 2 | 1 | 3.5767 | 0.597 |
| 7503_Test,3375 | K.VLSPQGDPWDSVAR.I | 1527.66322 | 0.72622 | 2 | 1 | 3.5194 | 0.5976 |
| 7503_Test,3381 | K.VLSPQGDPWDSVAR.I | 1527.66322 | 0.41922 | 2 | 1 | 3.7652 | 0.6401 |
| 7503_Test,3186 | R.IGEPLMLSCK.G | 1148.39198 | 1.34298 | 2 | 1 | 2.3798 | 0.2311 |
| 7503_Test,3207 | R.IGEPLMLSCK.G | 1148.39198 | -1.67902 | 2 | 1 | 3.6089 | 0.3363 |
| 7503_Test,3214 | R.IGEPLMLSCK.G | 1148.39198 | -1.85002 | 2 | 1 | 3.4448 | 0.329 |
| 7503_Test,2490 | R.RPLNTAPIQPR.V | 1263.47427 | 0.44327 | 2 | 1 | 2.6695 | 0.4368 |
| 7503_Test,2502 | R.RPLNTAPIQPR.V | 1263.47427 | 0.28727 | 2 | 1 | 2.631 | 0.4262 |
| 7503_Test,3936 | R.SELTVTPAQGGTTPTYSCSFSLGLPR.R | 2728.9858 | 0.3088 | 3 | 1 | 4.0758 | 0.546 |
| 7503_Test,3943 | R.SELTVTPAQGGTTPTYSCSFSLGLPR.R | 2728.9858 | -0.0362 | 2 | 1 | 4.3011 | 0.611 |
| 7503_Test,3945 | R.SELTVTPAQGGTTPTYSCSFSLGLPR.R | 2728.9858 | -0.4462 | 3 | 1 | 4.0494 | 0.5186 |
| 7503_Test,3946 | R.SELTVTPAQGGTTPTYSCSFSLGLPR.R | 2728.9858 | 0.2578 | 2 | 1 | 4.5026 | 0.6591 |
| 7503_Test,3951 | R.SELTVTPAQGGTTPTYSCSFSLGLPR.R | 2728.9858 | 1.4088 | 2 | 1 | 4.1054 | 0.6156 |
| 7503_Test,3676 | R.VYQIPGKPEIVNPASELTANVPNK.V | 2579.93287 | -0.88213 | 3 | 1 | 5.1792 | 0.5532 |
| 7503_Test,3681 | R.VYQIPGKPEIVNPASELTANVPNK.V | 2579.93287 | 1.05187 | 3 | 1 | 4.902 | 0.5076 |
| 7503_Test,3707 | R.VYQIPGKPEIVNPASELTANVPNK.V | 2579.93287 | 0.01887 | 2 | 1 | 4.7057 | 0.6362 |

**Supplemental Table 3 (b). Peptide sequences matched to RAGE (residues 1–342 correspond to the extracellular domain*) are shown below.**

| | | | | | | | | | |
|---|---|---|---|---|---|---|---|---|---|
| 10 | 20 | 30 | 40 | 50 | 60 | 70 | 80 | 90 | 100 |
| MPTGTVARAW | VLVLALWGAV | AGGQNITARI | GEPLMLSCKG | APKKPTQKLE | WKLNTGRTEA | WKVLSPQGDP | WDSVARILPN | GSLLLPAIGI | VDEGTFRCRA |
| 110 | 120 | 130 | 140 | 150 | 160 | 170 | 180 | 190 | 200 |
| TNRLGKEVKS | NYRVRVYQIP | GKPEIVNPAS | ELTANVPNKV | GTCVSEGSYP | AGTLSWHLDG | KPLIPDGKGT | VVKEETRRHP | ETGLFTLRSE | LTVTPAQGGT |
| 210 | 220 | 230 | 240 | 250 | 260 | 270 | 280 | 290 | 300 |
| TPTYSCSFSL | GLPRRRPLNT | APIQPRVREP | LPPEGIQLLV | EPEGGTVAPG | GTVTLTCAIS | AQPPPQIHWI | KDGTPLPLAP | SPVLLLPEVG | HEDEGIYSCV |
| 310 | 320 | 330 | 340 | 350 | 360 | 370 | 380 | 390 | 400 |
| ATHPSHGPQE | SPPVNIRVTE | TGDEGQAAGS | VDGSGLGTLA | LALGILGGLG | IAALLIGAIL | WRKRQPRLEE | RKAPESQEDE | EERAELNQSE | EAEMPENGAG |
| 402 | | | | | | | | | |
| GP | | | | | | | | | |

*Reference: Renard, C.; Chappey, O.; Wautier, M. P.; Nagashima, M.; Morser, J.; Scherrmann, J. M.; Wautier, J. L., The human and rat recombinant receptors for advanced glycation end products have a high degree of homology but different pharmacokinetic properties in rats. *J Pharmacol Exp Ther* **1999,** 290, (3), 1458-66.
